# Bioimpedance spectroscopy can precisely discriminate human breast carcinoma from benign tumors

**DOI:** 10.1097/MD.0000000000005970

**Published:** 2017-01-27

**Authors:** Zhenggui Du, Hangyu Wan, Yu Chen, Yang Pu, Xiaodong Wang

**Affiliations:** a Department of Breast Surgery; b Laboratory of Breast Disease; c Laboratory of Pathology, West China Hospital, Sichuan University, Chengdu, China.

**Keywords:** benign tumors, Bioimpedance spectroscopy, breast cancer, diagnostic value, frozen section

## Abstract

Intraoperative frozen pathology is critical when a breast tumor is not diagnosed before surgery. However, frozen tumor tissues always present various microscopic morphologies, leading to a high misdiagnose rate from frozen section examination. Thus, we aimed to identify breast tumors using bioimpedance spectroscopy (BIS), a technology that measures the tissues’ impedance. We collected and measured 976 specimens from breast patients during surgery, including 581 breast cancers, 190 benign tumors, and 205 normal mammary gland tissues. After measurement, Cole-Cole curves were generated by a bioimpedance analyzer and parameters *R*_0_/*R*_*∞*_, *f*_*c*_, and *α* were calculated from the curve. The Cole-Cole curves showed a trend to differentiate mammary gland, benign tumors, and cancer. However, there were some curves overlapped with other groups, showing that it is not an ideal model. Subsequent univariate analysis of *R*_*0*_/*R*_*∞*_, *f*_*c*_, and *α* showed significant differences between benign tumor and cancer. However, receiver operating characteristic (ROC) analysis indicated the diagnostic value of *f*_*c*_ and *R*_0_/*R*_*∞*_ were not superior to frozen sections (area under curve [AUC] = 0.836 and 0.849, respectively), and *α* was useless in diagnosis (AUC = 0.596). After further research, we found a scatter diagram that showed a synergistic effect of the *R*_0_/*R*_*∞*_ and *f*_*c*_, in discriminating cancer from benign tumors. Thus, we used multivariate analysis, which revealed that these two parameters were independent predictors, to combine them. A simplified equation, *RF*^′^ = 0.2*f_c_* + 3.6*R*_0_/*R*_∞_, based on multivariate analysis was developed. The ROC curve for RF′ showed an AUC = 0.939, and the sensitivity and specificity were 82.62% and 95.79%, respectively. To match a clinical setting, the diagnostic criteria were set at 6.91 and 12.9 for negative and positive diagnosis, respectively. In conclusion, RF′ derived from BIS can discriminate benign tumor and cancers, and integrated criteria were developed for diagnosis.

## 1 Introduction

Techniques including physical examination, ultrasonography, mammography, and fine needle aspiration biopsy sometimes cannot allow for an accurate diagnose of a patient with breast tumor. Local excision and subsequent pathological examination becomes the final option. As a result, frozen section diagnosis during surgery is very meaningful to both doctors and patients, guiding subsequent surgery steps and preventing patients from having to undergo a second surgery. However, even for an experienced pathologist, making an accurate diagnose from frozen sections is difficult, with an approximately 10% to 20% misdiagnosis rate.^[1–3]^ Thus, in some small medical centers or in some developing countries lacking well-trained pathologists, this can be a big challenge.^[4,5]^ Furthermore, intraoperative frozen section diagnosis is time-consuming, resulting in longer time spent in surgery. Therefore, finding other feasible and reliable ways is valuable in the clinic.

Different organs are different in structure, components, and ratio of water and lipids, which leads to different physicochemical features, such as impedance.^[6]^ When we apply an electrical potential to tissues, the impedances can be measured by the potential and current. Thus, we could utilize the impedances of tissues to distinguish them. The application of bioimpedance has long been investigated in medical areas to identify human tissues or detect invisible lesions, and its accuracy is relatively high.^[7–10]^ As to larger samples, we found that several studies published recently reported that BIS can detect breast cancer surgery related arm lymphedema at an earlier stage. In these studies, researchers put 2 measurement electrodes at 10 cm intervals along the dorsal surface of the patient's arm at locations of 10, 20, 30, and 40 cm from the ulnar styloid.^[11]^

With respect to cancer diagnosis, we realized that carcinomas are different from benign tumors in components, thereby resulting in different impedances. Some studies have already demonstrated that this method can discriminate carcinomas and benign tumors in tissues such as the lung and thyroid.^[12,13]^ However, few studies have reported its successful application in the clinic for breast diseases. Thus, we designed this study to investigate the diagnostic value of BIS in breast tumors.

## 2 Patients and methods

### 2.1 Patients

From September 2013 to September 2015, 1003 consecutive patients hospitalized at the Department of Breast Surgery, West China Hospital who did not receive core needle biopsy or fine needle aspiration were enrolled in the study, 27 patients who met the inclusion criterion did not sign the informed consent were excluded. Tumor tissues and mammary gland tissues collected from these patients were tested with impedance, and only tumors were examined by frozen section diagnosis during surgery. The pathology examination of the tumors was performed by the Department of Pathology after surgery. The study protocol was approved by the Ethics Committee of Sichuan University and relevant institutions for the use of human subjects in research. All women provided written informed consent.

### 2.2 Basic theory of BIS and measuring method

The impedances of tissue vary with the frequency of the current. A specific instrument can calculate the impedance from the voltage and current. In this study, we used a bioimpedance spectroscopy analyzer (Mscan1.0B, Sealand Technology, Chengdu, China) for measuring. The machine consists of 2 parts, the main part including the analyzer, liquid crystal display, a power supply system, and a measuring probe with 4 electrodes. One trained staff was responsible for all measurements and was blind for all informations of the patients. We collected specimens from breast surgery. Before measurement, we would correct the BIS machine using a standard resistor element, which gauged by a highly accurate laboratory machine to ensure accuracy. Specimens should be incised to get a smooth surface and the liquid on the surface should be wiped away to avoid breaking the circuit. The probe should keep stable when in contact with samples, because the pressure on samples, especially the soft ones, may alter the volume actually measured. The measured part was marked with sutures before being sent for frozen section and pathologic examination. Impedances of tissues under different frequencies were recorded and subsequently fitted to a Cole-Cole function curve using a Levenberg–Marquardt algorithm.

### 2.3 Parameters from the Cole-Cole curve

Four parameters, *R*_0_, *R*_*∞*_, *f*_*c*_, and *α*, were obtained from the Cole-Cole function curve equation: *R*^∗^(*f*) = *R*_∞_ + (*R*_0_ − *R*_∞_/1 + [*jf*/*f_c_*]^[1−α]^). *R*_0_ is the resistance under direct current, *R*_*∞*_ is the resistance under alternating current with maximum frequency, *f*_*c*_ is the frequency at highest reactance, and *α* is the dispersion coefficient. Because *R*_0_ and *R*_*∞*_ were both affected by the method of measurement and volume of tissue, we used *R*_0_/*R*_*∞*_ instead to eliminate such influences when analyzing.

### 2.4 Statistics

Descriptive statistics included means and ranges. Categorical data are presented as percentages. Continuous variables were compared using the unpaired Student *t* test. Multivariate analysis was performed to identify independent determinants for predicting carcinomas (logistic regression stepwise backward procedure). All statistical evaluations were performed using SPSS 18.0 for Windows software (IBM, Chicago, IL). For the receiver operating characteristic (ROC) curve analysis, we used MedCalc (version 12.0) to calculate the sensitivity, specificity, area under the curve, and to select the optimal cut-off value for diagnosis. Results with *P* values <0.05 were considered statistically significant.

## 3 Results

### 3.1 Patient characteristics and frozen section results

A total of 976 women were tested. The average age was 47.04 (18–83). Among them, 581 were breast carcinomas, 190 were benign tumors, and 205 were only tested for normal mammary gland tissue. The results of postoperative pathology examinations are listed in Table 1. Frozen section diagnosis was performed on each patient with a breast tumor during the surgery. The sensitivity of frozen section diagnosis was 92.1% (535/581), specificity was 98.9% (188/190), and accuracy was 93.8% (723/771).

**Table 1:**
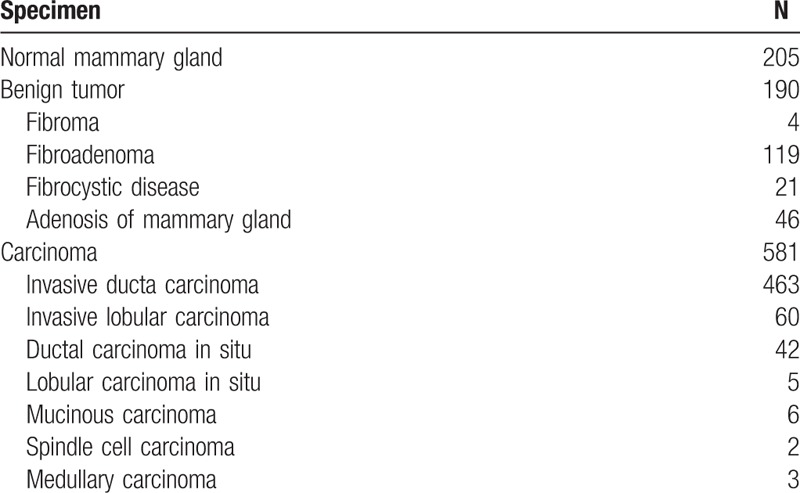
Pathology results of patients.

### 3.2 Cole-Cole curve of gland tissue, benign tumors, and breast cancer

**Figure 1:**
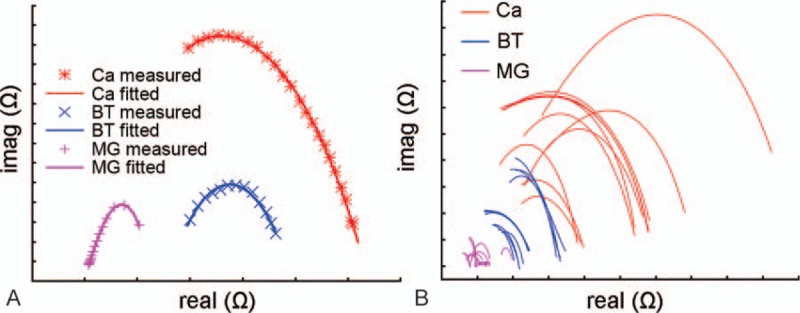
(A) Typical Cole-Cole curves of mammary gland tissue, benign tumor, and breast carcinoma. (B) Some Cole-Cole curves of benign tumors and breast carcinomas overlapped. BT = benign tumor, Ca = carcinoma, MG = mammary gland.

Initially, we tested dozens of samples and found that the Cole-Cole curves of the 3 types of tissues distributed approximately into 3 different areas on the coordinate axis. Typical curves show the potential diagnostic value of the model (Fig. 1A). However, after further measuring, we found that on the Cole-Cole curves, a small proportion of curves overlapped (Fig. 1B). No diagnostic criteria could be developed. Therefore, the Cole-Cole curves were not an ideal diagnostic model. A new method was needed to make it meaningful.

### 3.3 Diagnostic value of *R*_0_/*R*_*∞*_ and *f*_*c*_ in discriminating cancer and benign tumors

**Figure 2:**
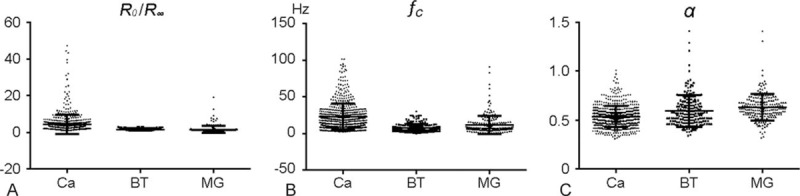
The differences of parameters, including *R*_0_/*R*_*∞*_ (A), *f*_*c*_ (B), and *α* (C), in the three groups. BT = benign tumor, Ca = carcinoma, MG = mammary gland.

We next focused on the parameters derived from the Cole-Cole curve. The data of *R*_0_/*R*_*∞*_, *f*_*c*_, and *α* are shown in Fig. 2(A–C). Analyses were performed between each group but discriminating gland tissue and tumors is meaningless in the clinic; thus, we mainly focused on diagnosing breast tumors. Univariate analysis showed that *R*_0_/*R*_*∞*_ (*P* < 0.001) and *f*_*c*_ (*P* < 0.001) were significantly higher in the cancer group than the benign tumor group, while *α* (*P* < 0.001) was lower.

**Figure 3:**
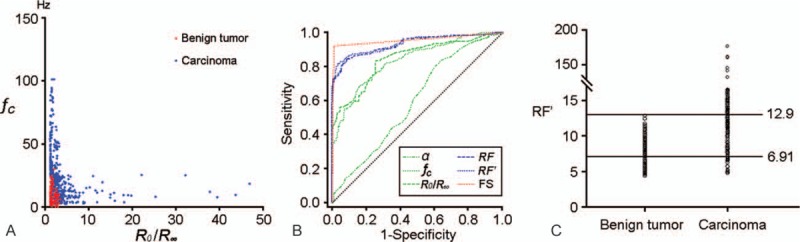
(A) Scatter diagram of *R*_0_/*R*_*∞*_ and *f*_*c*_ derived from breast tumors. (B) ROC curve for parameters *R*_0_/*R*_*∞*_, *f*_*c*_, *α*, RF, RF′, and frozen section diagnosis. (C) The diagnostic criteria for RF′ in the clinical setting. RF = regression factor, ROC = receiver operating characteristic.

To judge the diagnostic power of the parameters, we performed ROC curve analysis for each parameter to select the most useful criterion in the clinical setting. The areas under the ROC curves were 0.849 (95% confidence interval [CI], 0.822–0.873), 0.836 (95% CI, 0.808–0.861), and 0.596 (95% CI, 0.561–0.631) for *R*_0_/*R*_*∞*_, *f*_*c*_, and *α*, respectively. In contrast, the AUC of frozen section diagnosis was 0.955 (95% CI, 0.938–0.969) (Fig. 3B). The data indicated that *α* was useless in diagnosis even though it showed significant differences in univariate analysis, and *R*_0_/*R*_*∞*_ and *f*_*c*_ had better performance but were also obviously lower than the accuracy of frozen section diagnosis.

### 3.4 Combining *R*_0_/*R*_*∞*_ and *f*_*c*_ to form a new diagnostic model

On the scatter diagram consisting of *R*_0_/*R*_∞_ and *f*_c_, we found that benign tumors almost always existed in the bottom-left corner, apparently separated from cancers (Fig. 3A). This phenomenon indicated that combining these 2 parameters may have better diagnostic power. Multivariate analysis for *R*_0_/*R*_∞_, *f*_c_, and *α* revealed only *R*_0_/*R*_*∞*_ (*P* < 0.001) and *f*_*c*_ (*P* < 0.001) were independent parameters (Table 2). To combine them, a parameter based on the Exp value from multivariate analysis, which was named regression factor (RF), was generated and defined as: RF = 1.2^f_c_^ + 4.6*R*_0_/*R*_∞_. When parameter RF was analyzed with an ROC curve, we found that the area under the curve was 0.933 (95% CI, 0.913–0.950), indicating a higher predictive accuracy. We wanted to simplify the equation; therefore, an equivalent parameter RF′ was generated by a mathematics method, defined as: *RF*^′^ = 0.2*f_c_* + 3.6*R*_0_/*R*_∞_. In ROC curve analysis, the AUC of RF′ was 0.939 (95%CI, 0.919–0.955), cut-off value was 11.33, with a sensitivity and specificity of 82.62% and 95.79%, respectively (Table 3, Fig. 3B).

**Table 2:**
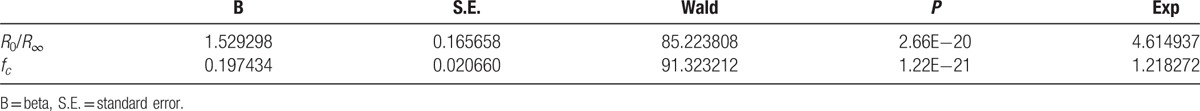
Multivariate analyses of parameters in Benign tumor group and Carcinoma group.

**Table 3:**
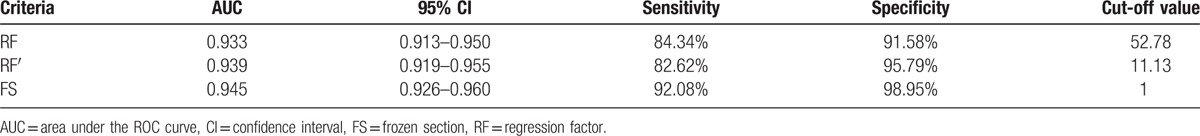
Diagnostic characteristics of RF, RF′, and frozen section diagnosis in discriminating breast tumors.

## 4 Discussion

In this study, we used BIS to measure the impedances of breast tissues, including mammary gland tissue, benign tumors, and carcinomas. A traditional Cole-Cole model was implemented to fit the measured impedances into a function curve. However, this model was meaningless in diagnosis because of the existence of overlapped curves. To select new criteria for discrimination of carcinomas and benign tumors, further research found that in scatter diagrams, parameters *R*_0_/*R*_*∞*_ and *f*_*c*_ exhibited a synergistic effect. Multivariate analysis also showed they were independent parameters. Thus, we combined them based on the Exp value to generate the equation RF = 1.2^*f_c_*^ + 4.6^*R*_0_/*R*_∞_^. The AUC for parameter RF was 0.933. To simplify it, another equivalent equation *RF*^′^ = 0.2*f_c_* + 3.6*R*_0_*R*_∞_ was generated, and the AUC for parameter RF′ was 0.939, and the sensitivity and specificity was 82.62% and 95.79%, respectively, at the cut-off value, which showed nearly equal diagnostic power as frozen sections.

Although the model RF′ showed high accuracy, when utilized in a clinical setting, detailed criteria should be established based on actual clinical requirements. If the diagnostic cut-off value was set at 11.13, we could obtain the largest sum of sensitivity (82.62%) and specificity (95.79%). For intraoperative pathology examination, low sensitivity is related to false-negative findings and low specificity is related to false-positive results, which respectively lead to reoperations and over-resection. For both patients and clinicians, requiring a second operation is more acceptable than over-treatment. Several studies have reported over-resection caused by false-positive frozen sections leads to many serious results, including more injuries, irreversible changes in appearance, and subsequent psychosocial effects.^[14–16]^ In other words, a high specificity is much more important than sensitivity. Thus, we could not set a cut-off value simply to obtain the highest accuracy.

In our diagnostic model, we found a trend that a higher value of RF′ resulted in a lower possibility of being a benign tumor. When the diagnostic criterion was set at 12.9, the specimens higher than it were all carcinomas, the specificity was 100%. However, pursuing that the highest specificity leads to lower sensitivity, the sensitivity at that criterion was 69.88%.

More than 30% of breast cancer patients require a second surgery. Reoperations obviously cost more time and money for patients, and consuming extra medical resources is also a big problem.^[17]^ Furthermore, clear margins are much difficult to obtain from a previously locally excised patient when performing breast-conserving surgery.^[18–20]^ Thus, such a false-negative rate is relatively high and unacceptable. Criterion to guarantee high sensitivity is important as well.

To form an integrated diagnose system, we applied a categorized strategy. Patients were divided into 3 groups: with an RF′ value higher than 12.9, a total of 406 (69.9% from 581 carcinomas) patients were all found to be carcinoma. At that criterion, the specificity was 100%. With an RF′ value lower than 6.91, only 29 (5% in 581 carcinomas) breast cancer patients existed in this group, meaning that fewer than 5% of breast cancer patients would require reoperation due to the false-negative results. Referring to the sensitivity of our frozen section results, we speculated that even an experienced pathologist can hardly exceed such accuracy.

When the RF′ value ranged from 6.91 to 12.9, 146 carcinomas (25.1% in 581 carcinomas) and 80 benign tumors (42.1% of 190 benign tumors) were not diagnosed by our model; therefore, for such patients, frozen section examination was still essential. Furthermore, even if they were not diagnosed by BIS, the 42.1% benign tumor patients were not suffering from any damages, as long as false-positive results were avoided completely, their pathology results can be obtained after surgery. We show the criteria in Fig. 3C.

In our hospital, the Department of Pathology is famous nationwide. During a surgery, 2 experienced pathologists are responsible for the frozen section diagnosis. Under such situations, the criteria we developed showed equivalent power to frozen sections. At some small medical centers lacking experienced pathologists, the method would have even more advantages. Finally, as compared with frozen section diagnosis, we can obtain the results in only a few minutes and thus plenty of time would be spared during surgeries.

Our study had some limitations. We tested an adequate number of patients to build the model, but more accurate criteria may need more specimens and multicenter trials. In addition, a verification test should be designed to judge the diagnostic power. Finally, the method was based on the intrinsic property of tumor tissues. Criteria-matching tumors with special types (e.g., mucinous carcinoma, medullary carcinoma) should be investigated.

## 5 Conclusions

The results of this study indicate that the parameter RF′ derived from BIS can discriminate breast tumors. Applying our diagnostic criteria, almost no benign tumors would be misdiagnosed and nearly 70% of cancer patients would be diagnosed correctly. Only around 5% of cancer patients would undergo a second operation but a quarter of cancer patients would still require frozen section diagnosis.
